# Effect of intradialytic progressive resistance exercise on physical fitness and quality of life in maintenance haemodialysis patients

**DOI:** 10.1002/nop2.585

**Published:** 2020-08-21

**Authors:** Fan Zhang, Liuyan Huang, Weiqiong Wang, Qiyun Shen, Huachun Zhang

**Affiliations:** ^1^ Nephrology Department Longhua Hospital Shanghai University of Traditional Chinese Medicine Shanghai China; ^2^ Hemodialysis Center Longhua Hospital Shanghai University of Traditional Chinese Medicine Shanghai China; ^3^ Department of Nursing Longhua Hospital Shanghai University of Traditional Chinese Medicine Shanghai China

**Keywords:** exercise, fitness, haemodialysis, nurses, nursing, quality of life

## Abstract

**Purpose:**

To investigate the impact of intradialytic progressive resistance exercise (IPRE) on physical fitness and quality of life in maintenance haemodialysis (MHD) patients.

**Methods:**

Subjects were allocated randomly to the exercise group received IPRE and the control group underwent a haemodialysis session alone. Outcomes measured were including physical fitness ascertained by 6‐min walk test, sit‐to‐stand 10 test and handgrip strength. Kidney Disease Quality of Life Instrument was used to assess the quality of life, and also recorded the adverse event at each exercise session.

**Results:**

A total of 87 patients were analysed: 43 in the exercise group and 44 in the control group. After 12 weeks, there were significant improvements in physical fitness and past of the dimension of the scale in the exercise group.

**Conclusions:**

IPRE can improve the physical fitness and quality of life in patients underwent MHD with no serious adverse events or safety issues.

## 1 INTRODUCTION

Patients with end‐stage renal disease (ESRD) need renal replacement therapy such as dialysis or transplantation for survival, of which maintenance haemodialysis (MHD) is the most widely used (USRDS, 2018). Protein‐energy wasting (PEW) is a major complication in MHD patients, with a prevalence rate of 15%–74% (Mazairac et al., 2011; Yasui et al., 2016). In skeletal muscle, PEW decreased the muscle protein synthesis and increased the rate of muscle proteolysis, resulting in muscle atrophy, and it adversely affects multiple patient‐centred outcomes, including lower physical fitness. This diminished physical fitness leads to a significant loss of independence with 95% of MHD patients not fully independent and seek assistance with at least one activity of daily living (ADL). Unfortunately, inability to independently complete ADLs is a independent predictor of MHD mortality (Perl et al., 2017).

Exercise is well tolerated among MHD patients and may be an effective strategy to prevent muscle atrophy in MHD patients. Clinical trials have shown that progressive resistance exercise can reserve muscle atrophy. This change is the basis for improving physical fitness (such as muscle strength) (Rosa et al., 2018; Watson, 2015), ADLs (O'Shea, Taylor, & Paratz, 2009) and QoL (Cheema, Chan, Fahey, & Atlantis, 2014; Jorge et al., 2015). Hence, the current guidelines encouraged to perform resistance exercise “at least two times per week on non‐consecutive days” (Smart et al., 2013). Despite this, most MHD patients do not meet the minimum level of physical activity compared with guidelines (Avesani et al., 2012). Among this population, 45% choosing sedentary, citing issues such as fatigue, perception of too many medical problems, too much trouble, lack of motivation and lack of time (Moorman et al., 2019).

The intradialytic progressive resistance exercise (IPRE) means exercise during haemodialysis session, with several major advantages: (a) patients can exercise under the supervision of medical staff and ensure patient safety; (b) patients can exercise during dialysis without extra time; and (c) it can improve dialysis adequacy. However, unlike patients in the USA and European countries (Olvera‐Soto, Valdez‐Ortiz, López Alvarenga, & Espinosa‐Cuevas, 2015), Chinese patients are usually lying in bed on dialysis, which precludes the application of the same IPRE. Therefore, as a strategy to prevent muscle atrophy in MHD patients, the effect of IPRE requires further verification. Therefore, this study aimed to determine whether an IPRE intervention could improve physical fitness and QoL in MHD patients.

## 2 METHODS

### 2.1 Study design

The purpose of this study was to investigate the impact of IPRE on physical fitness and quality of life in MHD patients using a single‐centre randomized control trial design.

### 2.2 Setting and sample

The patients were recruited from the haemodialysis centre of a hospital in Shanghai, China, between August–November 2018. Patients who met the following eligibility criteria were included: (a) diagnosed with ESRD and received haemodialysis therapy thrice weekly for at least 3 months; (b) 18 years of age or older; (c) non‐wheelchair bound; (d) able to provide informed consent in Chinese, and potential participants were excluded if they were: (a) diagnosed mental health disorder and (b) unstable cardiopulmonary disease. The sample size for the participated selection was calculated by using the formula: N_1_ = N_2_ = 2 [σ(t_α_ + t_β_)/(μ_1_−μ_2_)]^2^. The minimum sample size required for a two‐sided test with α = 0.05, β = 0.1 and t_0.05_ = 1.96, t_0.1_ = 1.282. According to Wu's study (Wu, He, Yin, Cao, & Ying, 2014), μ_1_−μ_2_ = 9.2, σ = 12.9 and 40 cases were obtained by calculation. Considering a 10% missing rate, finally, we planned to recruit 45 patients in each group.

They were allocated to the exercise group and the control group randomly by a computer (Random Number: 19,930,627). The study protocol was approved by the Ethics Committee of Longhua Hospital (approval number: 2017LCSY352), and written informed consent was obtained from all participants before the study.

### 2.3 Intervention

The IPRE was developed by the authors, based on the Life Option (Option, 2019), guidelines (Koufaki, Greenwood, Painter, & Mercer, 2015) and the previous studies (Olvera‐Soto et al., 2016), according to exercise prescription (Table 1).

1. Frequency: Considering the patient's tolerance, the frequency was as follows: twice a week in week 1–4, 2 sets of 8–10 repetitions of each movement and thrice a week in the next eight weeks, 3 sets of 11–12 repetitions;
2. Intensity: Exercises were performed at a rating of perceived exertion (RPE) of 10–13 (light to somewhat hard) on the Borg scale and RPE 8–10 for warm‐up and cool down.
3. Type: Resistance was applied to the wrist and ankle, respectively. In each haemodialysis session, 4 motor actions were performed (Table 2);
4. Time: Resistance exercise is completed within 1–2 hr after the beginning of each haemodialysis session, including 5 min of warm‐up (arm and lower limb lifting exercise without resistance), 30–40 min of exercise (including 20–30 min of exercise, 2–3 min of rest between each group) and 5 min of cool down (after exercises, passive stretching of the lower limbs was performed to facilitate recovery);
5. Monitoring: The heart rate and blood pressure of patients were monitored during exercise every 15 min. In case of severe fatigue (RPE > 15), chest pain, hypoglycaemia, dizziness, pallor, dyspnoea, arrhythmia, blood pressure instability and other reactions, stop the exercise immediately.

**TABLE 1:** Components of exercise programme

Stages	Frequency	Intensity (based on RPE)	Types	Time
Warm‐up	Week 1–4: twice a week Week 5–12: thrice a week	8 ~ 10	Stretching	5 min
Resistance exercise		10 ~ 13	Bicep curl; Shoulder flexion; Leg extensions; Straight leg raise	30 ~ 40 min
Cool down		8 ~ 10	Stretching	5 min

**TABLE 2:** Movement of exercise

Movement of exercise	Aim of the exercise	Exercise instructions	Legend
1. Bicep curl	Increase the strength of your bicep muscle at the front of your upper arm	Keep your elbow in a fixed position by your side. Face your palm/fist towards the roof. Bend your elbow as you lift your palm towards your shoulder. Slowly relax the tension and return your palm to the starting position.	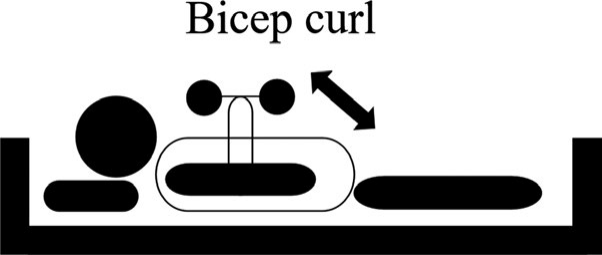
2. Shoulder flexion	Aims to increase the shoulder muscles to assist in your ability to lift and carry objects	Keeping your elbow straight, lift your arm towards the roof. Try to avoid “shrugging” your shoulder during the movement. Slowly return your arm to the starting position.	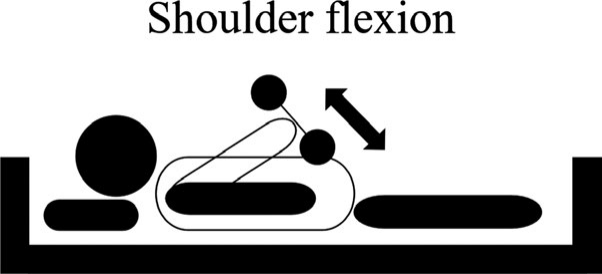
3. Leg extensions	Aim to strengthen the calf muscles. This assists in correct walking technique and balance	Keep one leg on the bed. Bend your knee to a 45‐degree position. Rest your heel on the bed, and point your toes forward as above. Alternate your legs for each repetition.	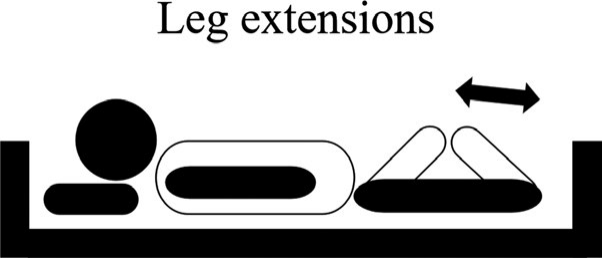
4. Straight leg raise	Improved lower body movements	Lift one leg off the chair, keeping the leg straight. Return your leg to the bed in a slow and controlled manner.	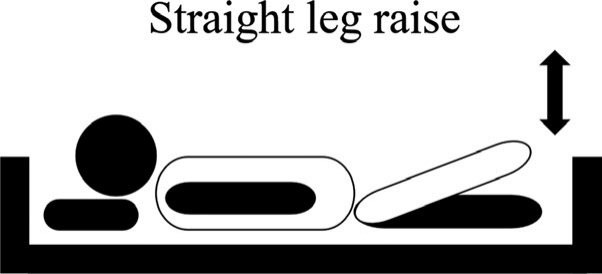

### 2.4 Study outcomes

The primary study outcome was physical fitness ascertained by 6‐min walk test (6MWT), sit‐to‐stand 10 test (STS 10) and handgrip strength (HGS). Secondary outcomes included quality of life and security. All outcomes were measured before and after the 12‐week intervention by a blinded assessor.

### 2.5 Physical fitness

The 6MWT was used to measure aerobic capacity, which required patients to turnaround on a flat, indoor corridor (30 m length) and measured the walking distance of 6 min. STS 10 was a method to evaluate individual functional mobility, of which subjects were asked to sit naturally in a 32 cm armless chair, relax hands and repeatedly stand up and sit down 10 times as fast as possible and recorded the time taken for it. HGS was assessed in non‐fistula hands using a dynamometer to assess patients’ upper limb muscle strength. Patients were first familiarized with the device and instructed to grip the dynamometer with maximum strength in a standing position with non‐fistula arms alongside the body. The highest value was recorded. The same haemodialysis nurse, who was trained by a physiotherapist, performed all physical fitness test.

### 2.6 Quality of life

Kidney Disease Quality of Life Instrument‐Short Form version 1.3 (KDQOL‐SF^TM^ v1.3), a 36‐item disease‐specific questionnaire, was employed to evaluate the patients’ quality of life. The questionnaire, consists of a 12‐item Short Form Health Survey (SF‐12) as a generic component for quality of life, was aggregated into two component summaries: physical component summary (PCS) and mental component summary (MCS), and 24 disease‐specific items were divided into three subscales: burden of kidney disease, symptom/problem and effects of kidney disease. Score was ranged from 0–100, with the higher of each dimension indicating a better quality of life. The Chinese version of the KDQOL‐36^TM^ has demonstrated acceptable levels of internal consistency (Cronbach's α = 0.69–0.78) and test–retest reliability (ICC = 0.70–0.86) (Tao, Chow, & Wong, 2014).

### 2.7 Adverse event

An adverse event was collected at each exercise session via interview from patients and by checking dialysis treatment records. Typically, adverse event like musculoskeletal injuries, cardiovascular events, intradialytic hypertension and access complications was recorded as they occurred, during the trial period. Decisions about whether events were attributable to the intervention were made by clinicians.

### 2.8 Data analysis

All data were analysed using SPSS software version 21.0 (SPSS Inc.), and a normality test was performed for continuous variables. For the descriptive analysis of all variables, those with a normal distribution were expressed as mean (*SD*), if not, expressed as the median and interquartile range. Categorical variables were summarized with frequencies and percentages. For both groups, comparisons within the group were evaluated with the paired‐samples Student's *t* test, respectively. In the case of variables with a non‐normal distribution, a Mann–Whitney *U* test was used, whereas a chi‐squared test was used for categorical variables. *p* < .05 was considered statistically significant.

## 3 RESULTS

### 3.1 Baseline characteristics

A total of 90 patients were initially enrolled in the study and were divided randomly into the exercise group (

**FIGURE 1:**
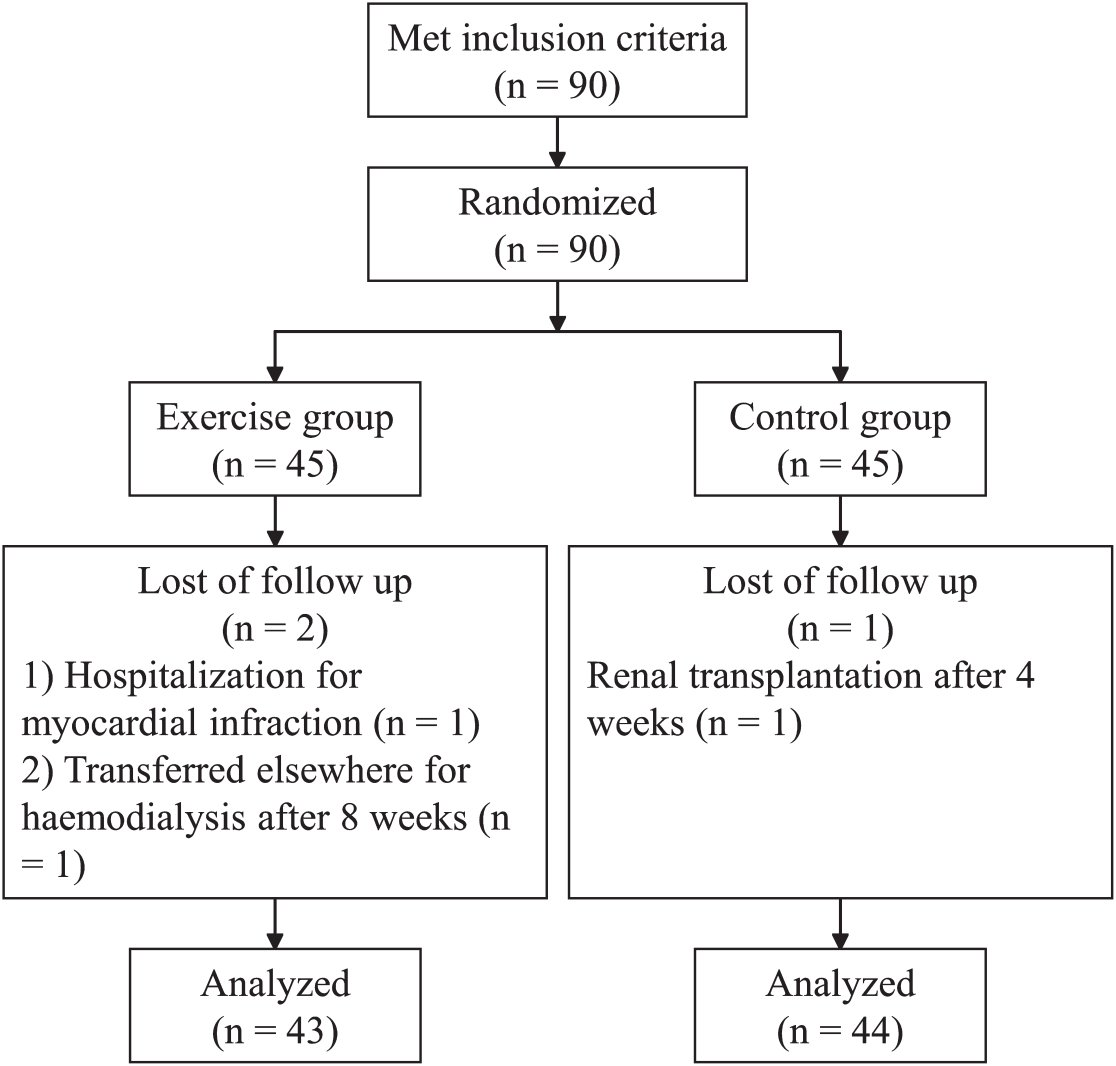
A total of 90 patients were initially enrolled in the study and were divided randomly into the exercise group (*n* = 45) and the control group (*n* = 45). Three patients were withdrawn from the study: in the exercise group, one patient was hospitalized for myocardial infarction and one patient transferred to another centre for haemodialysis; in the control group, one patient underwent renal transplantation. Therefore, 87 patients completed the whole study

*N* = 45) and the control group (*N* = 45). Three patients were withdrawn from the study: in the exercise group, one patient was hospitalized for myocardial infarction and one patient transferred to another centre for haemodialysis; in the control group, one patient underwent renal transplantation (Figure 1). Therefore, 87 patients completed the whole study, giving a completion rate of 96.7%.

The characteristics of the study participants are shown in Table 3. Of the 87 patients, 53 were male and 34 were female, with a mean age of 58.32 (SD12.42) years, and the median time on haemodialysis was 32.0 months. After randomization and before the intervention, both groups were statistically homogeneous (*p* > .05).

**TABLE 3:** Baseline characteristic of both groups (*n* = 87)

Variables	Total (*n* = 87)	Exercise group (*n* = 43)	Control group (*n* = 44)	Statistic	*p*
Age (years)	61.0 (51.0, 66.0)	60.0 (51.0, 66.0)	62.0 (54.0, 68.0)	−1.032^a^	.302
Time on dialysis	32.0 (20.0, 85.0)	39.0 (23.0, 89.0)	30.5 (19.3, 78.8)	−0.539^a^	.590
BMI	22.65 ± 3.00	22.80 ± 2.94	22.51 ± 3.08	−0.436^b^	.664
Gender				0.125^c^	.724
Male	53 (60.9%)	27 (50.9%)	26 (49.1%)		
Female	34 (39.1%)	16 (47.1%)	18 (52.9%)		
Highest education				0.413^c^	.938
Primary school or below	3 (3.4%)	2 (4.7%)	1 (2.3%)		
Junior high school	36 (41.4%)	18 (41.9%)	18 (40.9%)		
Senior high school	31 (35.6%)	15 (34.9%)	16 (36.4%)		
University or above	17 (19.5%)	8 (18.6%)	9 (20.5%)		
Primary cause				6.125^c^	.190
Glomerulonephritis	41 (47.1%)	22 (51.2%)	19 (43.2%)		
Diabetes mellitus	14 (16.1%)	3 (7.0%)	11 (25.0%)		
Hypertension	7 (8.0%)	5 (11.6%)	2 (4.5%)		
IgA nephropathy	8 (9.2%)	4 (9.3%)	4 (9.1%)		
Other	17 (19.5%)	9（20.9%)	8 (18.2%)		
Employment				1.655^c^	.437
In business	6 (6.9%)	4 (9.3%)	2 (4.5%)		
Retire	75 (86.2%)	35 (81.4%)	40 (90.9%)		
Unemployed	6 (6.9%)	4 (9.3%)	2 (4.5%)		
Financial status				7.137^c^	.068
Poor	29 (33.3%)	18 (41.9%)	11 (25.0%)		
Moderate	42 (48.3%)	16 (37.2%)	26 (59.1%）		
Good	13 (14.9%)	6 (14.0%)	7 (15.9%)		
Very good	3 (3.4%)	3 (7.0%)	0(0.0%)		

Note

Values are presented as the mean ± standard deviation, medians (interquartile ranges).

Nominal variables are presented as case number (percentage).

Abbreviation: BMI, body mass index.

^a^ *Z* value.

^b^ *t* value.

^c^ χ^2^ value.

### 3.2 Physical fitness

After the 12 weeks of intervention, the baseline outcomes were compared with the finals for each group. With this analysis, the group performing the IPRE showed beneficial and statistically significant changes in all three physical fitness variables. The higher this value, the worse the STS 10. The result signify physical fitness was diminished in the control group. The results are shown in Table 4.

**TABLE 4:** Comparison of the baseline and 12 weeks for the exercise and control group (*n* = 87)

Variables	Exercise group (*n* = 43)				Control group (*n* = 44)			
	Baseline	12 weeks	*t* or *Z*	*p*	Baseline	12 weeks	*t* or *Z*	*p*
Physical fitness								
6MWT	406.54 ± 85.61	409.49 ± 88.27	2.314^a^	.026	373.57 ± 89.63	373.21 ± 91.30	−0.436^a^	.665
STS 10	25.20 (23.40, 26.70)	23.80 (23.10, 25.70)	−4.041^b^	<.001	25.80 ± 2.06	26.40 ± 2.59	2.274^a^	.028
HGS	25.71 ± 8.48	26.57 ± 8.43	3.141^a^	.003	22.47 ± 7.01	22.03 ± 7.09	−1.395^a^	.170
Quality of life								
Burden of kidney disease	31.25 (18.75, 50.00)	50.00 (37.50, 56.25)	−2.555^b^	.011	40.63 (18.75, 56.25)	25.00 (18.75, 31.25)	−2.935^b^	.003
Symptom/Problem	79.17 (68.75, 87.50)	83.33 (75.00, 87.50)	−1.353^b^	.176	84.38 (71.87, 89.58)	77.08 (68.75, 81.25)	−2.245^b^	.025
Effect of kidney disease	56.25 (50.00, 65.63)	68.75 (56.25, 75.00)	−3.864^b^	<.001	62.50 (50.00, 71.10)	56.25 (50.00, 62.50)	−1.474^b^	.140
Physical component summary	39.32 ± 9.14	40.31 ± 8.74	0.608^a^	.547	35.16 ± 8.80	33.77 ± 7.55	−0.873^a^	.387
Mental component summary	51.76 (44.46, 56.87)	49.83 (46.54, 53.01)	−1.425^b^	.154	49.93 ± 8.02	43.85 ± 7.65	−3.632^a^	.001

Note

Values are presented as the mean ± standard deviation, medians (interquartile ranges).

Abbreviation: 6MWT, 6 min walk test; STS 10, sit‐to‐stand 10 test; HGS, handgrip strength.

^a^ *t* value.

^b^ *Z* value.

### 3.3 Secondary outcomes

Compared with the baseline, participants in the exercise group improve their “burden of kidney disease” with the score rise from (35.90 *SD* 23.47)–(45.64 *SD* 17.49) (*p* = .011) and “effect of kidney disease” with the score rise from (56.18 *SD* 12.36)–(65.19 *SD* 11.65) (*p* < .001). For the control group, the QoL score was significantly lower. The change was reflected in the significantly decrease of “burden of kidney disease,” “symptom/problem” and “mental component summary.” The results are shown in Table 4.

### 3.4 Adverse event

In terms of security, expected adverse events reported in exercise and control group, respectively, were musculoskeletal (muscle soreness: 4 vs. 1; cramps: 3 vs. 0), hypotension (3 vs. 3) and palpitations (1 vs. 1). No life‐threatening adverse event was observed during the trial, and there were no significant differences in the incidence of an event between the groups (Table 5).

**TABLE 5:** Adverse events observed in MHD patients in both groups

Adverse event	Exercise group	Control group	χ^2^	*p*
Muscle soreness	4 (9.3%)	1 (2.3%)	2.929	.087
Hypotension	3 (7.0%)	3 (6.8%)		
Cramps	3 (7.0%)	0 (0.0%)		
Palpitations	1 (2.3%)	1 (2.3%)		
Total	11 (25.6%)	5 (11.4%)		

Note

Nominal variables are presented as case number (percentage).

## 4 DISCUSSION

Muscle atrophy is highly prevalent in patients underwent MHD and is a marker of poor physical fitness in this population (Matsuzawa & Roshanravan, 2018), which was strongly associated with mortality and lower QoL. Our results showed the positive effects that thrice weekly, 50‐min sessions of IPRE on the physical fitness and improved quality of life for MHD patients.

Lower physical fitness is closely related to dialysis‐related fatigue, malnutrition, micro‐inflammatory and anaemia (Cobo et al., 2015). Noted that, poor physical fitness causes MHD patients being in a “physical inactivity” lifestyle. A sedentary lifestyle is the main reason for the further decline of physical fitness in MHD patients. These conclusions have also been confirmed in the present study, patients in the control group maintained a “no exercise” status, and all physical fitness variables have a trend of deterioration. Among them, a significant increase of STS 10 may explain the decrease of physical fitness caused by muscle atrophy in MHD patients with lack of exercise.

For 6MWT, a significant increase in walking distance was consistent with the results of several previous studies, which ranged from 20–48.5 m (Rosa et al., 2018; Segura‐Ortí, Kouidi, & Lisón, 2009; Wu et al., 2014). The difference level of improvement may be due to the low resistance load applied in this study, while the result of walking distance can be attributed to the morphology and function of skeletal muscle and the adaptability of nerve. Also, some studies did not observe a statistically significant increase in the 6MWT after exercise (Cheema et al., 2007; Kirkman et al., 2014; Vince, Julie, Tom, & Clase, 2002). Part of the reason for this difference is that the baseline values of the participants in these studies are higher. 6MWT is an important index to evaluate individual aerobic capacity. Multiple meta‐analyses showed that resistance exercise had a significant effect on improving 6MWT of MHD patients (Gomes, de Lacerda, Lopes, Martinez, & Saquetto, 2018; Matsuzawa et al., 2017), and this study also confirmed this result.

STS 10 is a method to evaluate lower limb muscle endurance in dialysis patients (Manfredini & Lamberti, 2014). Previous studies reported that the “sit‐to‐stand” test showed unsatisfactory results in MHD patients (Kim et al., 2014). The changes of STS 10 were noted as late as 12 weeks after the intervention. This result is consistent with others (Headley et al., 2002; Segura‐Ortí et al., 2009), indicating that a possible added benefit to IPRE intervention for MHD patients is that may improve lower limb muscle endurance.

In this study, HGS of 87 patients were (24.07 *SD* 7.90) kg, significantly lower than the average of healthy people in China (male: 65 kg, female: 30 kg) (Zhang, Cao, Gao, & Gong, 2011). The previous study showed that low grip strength was an independent risk factor for all‐cause mortality in MHD patients (Matos et al., 2014; Peng et al., 2015). Our study showed that HGS in the exercise group increased (0.86 *SD* 1.79) kg (*p* = .003), similar to Martin (Martinalemañy et al., 2016) and Olvera et al (Olvera‐Soto et al., 2016). HGS is a powerful index to evaluate the muscle strength of the upper limb. The study had confirmed that HGS would decrease gradually with the elderly. Evidence shows that resistance exercise is an effective strategy to maintain or even improve the HGS of healthy people and patients with chronic diseases (Abe, Thiebaud, & Loenneke, 2016).

The QoL in MHD patients is universally low and is influenced by various factors such as dialysis‐related symptoms, financial and work status (Weisbord, 2016). The KDQOL‐36^TM^ is an international instrument with high reliability and validity that has developed to evaluate QoL in patient who underwent dialysis (Tao et al., 2014). In the present study, patients who in the control group remained “sedentary” had lower scores in all dimensions. This result confirms the view of Perl (Perl et al., 2017) and Wu (Wu et al., 2004), QoL of MHD patients is lower, and the score of each dimension further decreases over time. Compared with baseline, there were differences in the exercise group in the KDQOL‐36^TM^ scores for burden of kidney disease and effect of kidney disease, but no observed change in PCS, which is consistent with Chen et al. used SF‐36 (Chen et al., 2010). On the contrary, a study of 40 participants in South Korea found that resistance exercise during dialysis three times a week significantly improved the scores of MCS and PCS evaluated by SF‐36. These differences may be due to the physical fitness obtained by the scale belongs to self‐report with a certain degree of subjectivity. The improvement of QoL may be explained as resistance exercise can reduce the symptoms of fatigue, sleep disorders and dyspnoea caused by kidney disease and enhance the ability of self‐management in life. Otherwise, the practical benefit for MHD patients got from exercise is the reduction of various risk factors, such as intradialytic hypotension, improving the inflammatory status, reducing the risk of cardiovascular disease.

No serious exercise‐related adverse were observed during the study. A patient in the exercise group withdrawn for myocardial infarction was not caused by exercise. However, during the study, four patients had muscle soreness and three patients had cramps in the exercise group, which may be caused by long‐term sedentary and unaccustomed exercise. Hypotension occurred in 3 patients and palpitation in 1 case, which may be caused by the frequent movement during exercise, and this kind of phenomenon did not occur after reducing the frequency. A small sample meta‐analysis showed that there was no significant difference in adverse events between the intradialytic exercise group and the control group (*p* > .11) (Sheng et al., 2014). Intradialytic exercise is carried out under the supervision of medical staff, which not only ensures safety but also improves compliance.

### 4.1 Limitations

The present study still has some limitations. First, the age and time on dialysis range of participants were relatively large, which might have affected the results. Second, the study lacked the psychological state assessment of MHD patients. The exercise psychology, especial self‐efficacy, may affect the patients’ behaviour and motivation. Third, only 6MWT, STS 10 and HGS were selected for physical fitness assessment, and there are number of other functions, including balance ability and body composition, that need to be further explored.

## 5 CONCLUSIONS

In conclusion, IPRE improved the physical fitness and quality of life in patients who underwent haemodialysis with no serious adverse events or safety issues were observed. Our findings suggest that this exercise modality could, therefore, be used as a strategy to reverse muscle atrophy in MHD patients. Despite the proven benefits of exercise interventions, it still is not part of routine care in many centres. In recent years, interventions using accelerometers or pedometers to promote physical activity in MHD patients have developed. Future research should focus on the potential cost benefit of these wearable sensors to change sedentary lifestyle in MHD patients.

## CONFLICT OF INTEREST

The authors have no conflicts of interest.

## AUTHOR CONTRIBUTIONS

Fan Zhang: Data analysis and write the article. Weiqiong Wang and Liuyan Huang: Data collection. Qiyun Shen: Patient recruitment. Huachun Zhang: Study design. In addition, all authors read and approved the final manuscript.
